# Longitudinal Transcriptome Analysis Reveals a Sustained Differential Gene Expression Signature in Patients Treated for Acute Lyme Disease

**DOI:** 10.1128/mBio.00100-16

**Published:** 2016-02-12

**Authors:** Jerome Bouquet, Mark J. Soloski, Andrea Swei, Chris Cheadle, Scot Federman, Jean-Noel Billaud, Alison W. Rebman, Beniwende Kabre, Richard Halpert, Meher Boorgula, John N. Aucott, Charles Y. Chiu

**Affiliations:** a Department of Laboratory Medicine, University of California, San Francisco, San Francisco, California, USA; b Department of Medicine, Johns Hopkins University School of Medicine, Baltimore, Maryland, USA; c Department of Biology, San Francisco State University, San Francisco, California, USA; d Qiagen Bioinformatics, Redwood City, California, USA

## Abstract

Lyme disease is a tick-borne illness caused by the bacterium *Borrelia burgdorferi*, and approximately 10 to 20% of patients report persistent symptoms lasting months to years despite appropriate treatment with antibiotics. To gain insights into the molecular basis of acute Lyme disease and the ensuing development of post-treatment symptoms, we conducted a longitudinal transcriptome study of 29 Lyme disease patients (and 13 matched controls) enrolled at the time of diagnosis and followed for up to 6 months. The differential gene expression signature of Lyme disease following the acute phase of infection persisted for at least 3 weeks and had fewer than 44% differentially expressed genes (DEGs) in common with other infectious or noninfectious syndromes. Early Lyme disease prior to antibiotic therapy was characterized by marked upregulation of Toll-like receptor signaling but lack of activation of the inflammatory T-cell apoptotic and B-cell developmental pathways seen in other acute infectious syndromes. Six months after completion of therapy, Lyme disease patients were found to have 31 to 60% of their pathways in common with three different immune-mediated chronic diseases. No differential gene expression signature was observed between Lyme disease patients with resolved illness to those with persistent symptoms at 6 months post-treatment. The identification of a sustained differential gene expression signature in Lyme disease suggests that a panel of selected human host-based biomarkers may address the need for sensitive clinical diagnostics during the “window period” of infection prior to the appearance of a detectable antibody response and may also inform the development of new therapeutic targets.

## IMPORTANCE

Lyme disease is the most common tick-borne infection in the United States, and some patients report lingering symptoms lasting months to years despite antibiotic treatment. To better understand the role of the human host response in acute Lyme disease and the development of post-treatment symptoms, we conducted the first longitudinal gene expression (transcriptome) study of patients enrolled at the time of diagnosis and followed up for up to 6 months after treatment. Importantly, we found that the gene expression signature of early Lyme disease is distinct from that of other acute infectious diseases and persists for at least 3 weeks following infection. This study also uncovered multiple previously undescribed pathways and genes that may be useful in the future as human host biomarkers for diagnosis and that constitute potential targets for the development of new therapies.

## INTRODUCTION

Lyme disease, a systemic tick-borne infection caused by the bacterial spirochete *Borrelia burgdorferi*, is the most common vector-borne disease in the United States and Europe (1). Over 30,000 cases in the United States are reported annually to the Centers for Disease Control and Prevention (CDC) (http://www.cdc.gov/lyme/stats/humancases.html). However, actual prevalence estimates are at least 10 times as high because of underreporting of cases and overreliance on insensitive diagnostic tests in the acute phase of infection (2). Lyme disease has been associated with arthritis, meningitis, facial palsy, and (rarely) myocarditis resulting in sudden death (3). Most patients treated with appropriate antibiotics recover rapidly and completely, but a minority of patients develop persistent symptoms correlating with disseminated disease, a greater severity of illness at presentation, and delayed antibiotic therapy (4). The proportion of Lyme disease patients with persistent symptoms varies greatly, from 0 to 50%, depending on the cohort of interest and the case definition used (4, 5). When lingering or recurrent symptoms are associated with a functional decline and persist for greater than 6 months, patients are considered to meet clinical criteria for post-treatment Lyme disease syndrome (PTLDS) (6), although the exact molecular mechanisms underlying this condition remain unknown.

Control of *B. burgdorferi* infection in early Lyme disease requires both innate and adaptive immune responses (7). Phagocytes constitute the first line of defense, engulfing the spirochete and producing Th1-type proinflammatory cytokines. Spirochetal lipoproteins can directly stimulate the B-cell response, and both lipidated and nonlipidated proteins trigger T-cell-dependent humoral responses. Decreased Th1 and increased Th17 responses have also been shown to play a role in the development of post-treatment Lyme disease symptoms during the chronic phase of the illness (8, 9). However, with the exception of antibiotic-refractory Lyme arthritis, very few studies have looked at the molecular mechanisms underlying persistent symptomatology in treated Lyme disease patients, and all to date have used targeted approaches assaying specific cytokine levels (8–10). The overall global and temporal pathways involved in human clinical infection with *B. burgdorferi* remain to be elucidated.

In this study, we applied next-generation sequencing of peripheral blood mononuclear cells (PBMCs) to investigate the transcriptomes of 29 patients with acute Lyme disease longitudinally from the time of diagnosis to 6 months post-treatment and those of 13 matched controls. We performed network and pathway analyses in order to gain insights into the molecular mechanisms underpinning acute Lyme disease and post-treatment symptoms and to discover potential diagnostic biomarkers.

## RESULTS

### Patient enrollment, sample collection, and transcriptome analysis.

**FIG 1:**
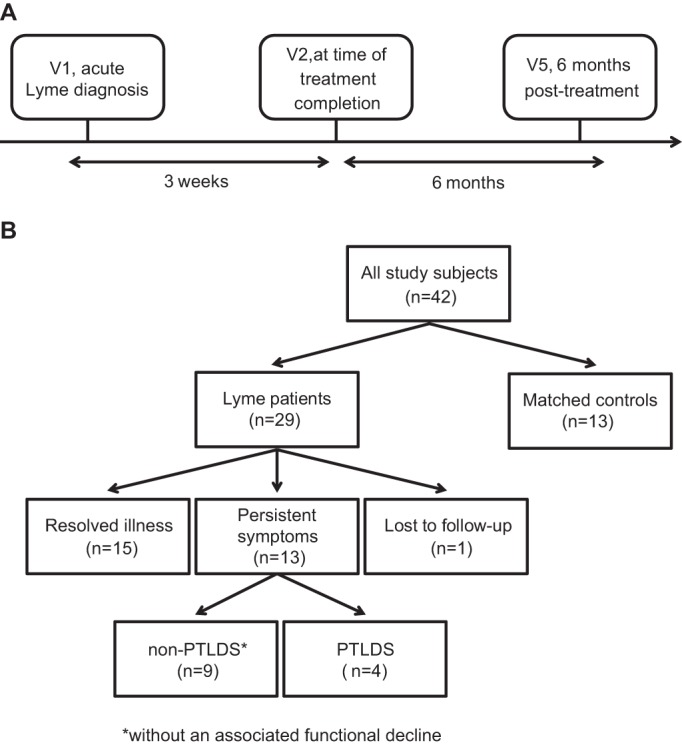
Schematic description of study design. (A) Timeline of clinical evaluation and PBMC sampling. (B) Flowchart of the number of patients with resolved illness or persistent symptoms. Abbreviations: non-PTLDS, post-treatment Lyme disease symptoms and no functional decline; PTLDS, PTLDS with a functional decline.

This study included a cohort of 29 patients with acute Lyme disease and 13 matched controls without acute illness. Transcriptome profiling by RNA sequencing (RNA-Seq) and pathway analysis were performed with PBMC samples collected at three time points, V1 (time of acute Lyme disease diagnosis and prior to starting antibiotic therapy), V2 (immediately after the completion of a 3-week course of doxycycline treatment), and V5 (6 months after the completion of therapy) (Fig. 1). Approximately 73 (± 43 [standard deviation]) million reads were generated per sample, and on average, 64.9% of the genes had nonzero counts (see Fig. S1 in the supplemental material).

No significant differences in age, sex, ethnicity, or preexisting comorbidities were noted between Lyme disease patients and controls (Table 1). Two-tiered antibody testing for Lyme disease with whole-cell lysates was positive in 20 (71.4%) of 28 patients tested, with 14/28 (50%) patients testing positive at the pretreatment visit and an additional 6/28 (21.4%) seroconverting during treatment (Table 1). The 29 Lyme disease patients were enrolled in a single season at the same geographic location, an outpatient clinic in suburban Maryland. At the 6-month follow-up visit (V5), 15 patients had fully recovered from the infection while 13 experienced persistent symptoms post-treatment, defined as new-onset fatigue, widespread musculoskeletal pain involving ≥3 joints, and/or cognitive dysfunction (11); 1 patient was lost to follow-up. Of the 13 patients with persistent symptoms, 4 were diagnosed with PTLDS on the basis of a recently proposed standardized case definition that included a documented functional decline at 6 months as a key criterion (6).

**TABLE 1:** Demographic and clinical characteristics of 29 patients with early Lyme disease and 13 matched controls^a^

Variable	Lyme disease patients^e^	Controls^f^	*P* value^g^
Avg age (yr)	52 (36–61) [20–71]	50 (42–61) [22–70]	0.62
Females	10/29 (34.5)	8/13 (61.5)	0.22
Non-Hispanic Caucasians	27/29 (93.1)	12/13 (92.3)	0.67
≥1 comorbidities	11/29 (38.9)	6/13 (46.2)	0.62
Carpal tunnel syndrome, mo	2/29 (6.9)	1/13 (7.7)	0.41
Diabetes, mo	0/29 (0.0)	2/13 (14.8)	
Heart disease, mo	4/29 (13.8)	1/13 (7.7)	
Lung disease, mo	1/29 (3.4)	0/13 (0.0)	
Migraines, mo	4/29 (13.8)	3/13 (23.1)	
Thyroid disease, mo	3/29 (10.3)	1/13 (7.7)	
Two-tier serology^b^			NA^h^
Pretreatment positive	14/28 (50.0)	0/13 (0.0)	
Seroconverted during treatment	6/28 (21.4)	0/13 (0.0)	
Negative	8/28 (28.6)	13/13 (100)	
Recovery status at V5			NA
Resolved	15/28 (53.6)	NA	
Persistent symptoms	13/28 (46.4)	NA	
Non-PTLDS^c^	9/28 (32.1)	NA	
PTLDS^d^	4/28 (14.3)	NA	
Lost to follow-up	1/29 (2.8)	NA	
Sampling season			
V1			<0.00001
Spring	3/29 (10.3)	3/13 (23.1)	
Summer	24/29 (82.8)	1/13 (7.7)	
Autumn	2/29 (6.9)	1/13 (7.7)	
Winter	0/29 (0.0)	8/13 (61.5)	
V2			<0.00001
Spring	1/28 (3.6)	3/13 (23.1)	
Summer	22/28 (78.6)	1/13 (7.7)	
Autumn	5/28 (17.9)	1/13 (7.7)	
Winter	0/28 (0.0)	8/13 (61.5)	
V5			0.023
Spring	7/28 (25.0)	3/13 (23.1)	
Summer	0/28 (0.0)	1/13 (7.7)	
Autumn	1/28 (3.6)	1/13 (7.7)	
Winter	20/28 (71.4)	8/13 (61.5)	

a Number/total (%) reported for categorical variables and median, IQR interquartile range (in parentheses), and range (in brackets) presented for continuous variables.

b One patient missing two-tier serology data.

c Non-PTLDS (persistent symptoms with no functional decline).

d PTLDS (persistent symptoms with functional decline).

e n = 29.

f n = 13.

g Lyme disease patients versus controls.

h NA, not applicable.

Six (40%) of the 15 patients with resolved illness and 6 (46%) of the 13 with persistent symptoms presented with early disseminated disease consisting of multiple erythema migrans (EM) lesions at the time of diagnosis (see Table S1 in the supplemental material). The average duration of acute illness, defined as the time from onset of EM rash and/or influenza-like symptoms to study enrollment and initiation of doxycycline therapy, was significantly longer in patients developing persistent symptoms (9.7 days for non-PTLDS and 19.3 days for PTLDS) than in patients with resolved illness (5.2 days) (*P* < 0.036) (see Table S1 in the supplemental material). In addition, the number of symptoms was significantly higher at all time points in patients with persistent symptoms than in those with resolved illness (*P* < 0.04) (see Table S1 in the supplemental material).

### Lyme disease gene expression signature.

**FIG 2:**
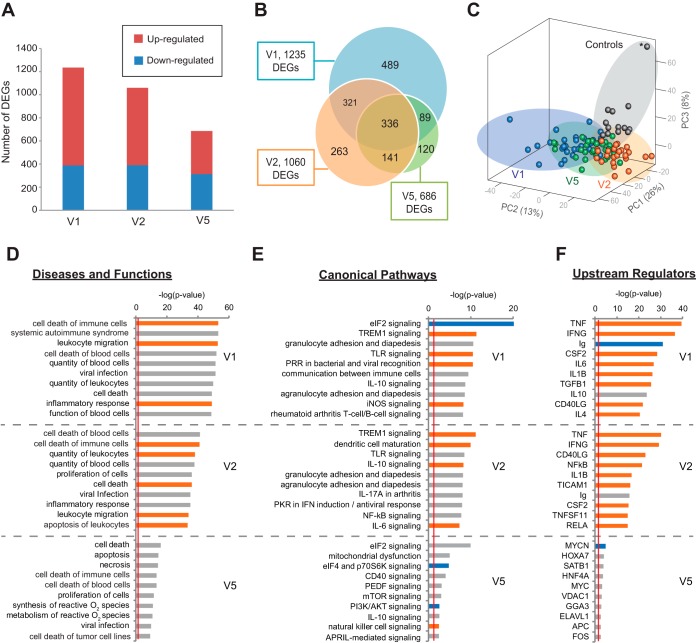
Longitudinal differential gene expression and pathway analysis of Lyme disease. (A) Bar chart of the numbers of genes found to be upregulated or downregulated at Lyme disease diagnosis (V1), 3 weeks post-treatment (V2, after a standard course of antibiotics), and 6 months post-treatment (V5). (B) Venn diagram representing the number of DEGs between Lyme disease patients and controls at three time points. (C) Principal component analysis (PCA) of Lyme disease patients and controls at three time points on the basis of 1,759 unique DEGs identified at V1, V2, and V5. The asterisk represents a subject in the control group who looks like an outlier in the PCA plot but is not shown to be an outlier by PCA analysis of the control samples (see Fig. S2 in the supplemental material). Note that the PC3 axis in the PCA plot accounts for only 8% of the variance in the data set. (D to F) Top 10 disease and functional categories (D), top 10 canonical pathways (E), and top 10 upstream regulators (excluding drug categories) (F) predicted to be involved in Lyme disease at (V1, V2, and V5) with categories, pathways, and genes ranked by the negative log of the *P* value of the enrichment score. The color scheme is based on *Z* scores, with activation in orange, inhibition in blue, and undetermined directionality in gray. The red line represents the designated significance threshold (*P* < 0.05).

We initially compared the transcriptomes of 29 Lyme disease patients at the time of diagnosis (V1) with those of 13 matched controls. This analysis revealed a total of 1,235 differentially expressed genes (DEGs) (Fig. 2A; Table 2). Approximately 69% (*n* = 847) of the DEGs were upregulated, and 31% (*n* = 388) were downregulated (Fig. 2A). Three weeks after diagnosis (V2), at the time of completion of a standard course of antibiotic treatment, 1,060 DEGs were found in both Lyme disease patients and controls, with 63% (*n* = 670) upregulated and 37% (*n* = 390) downregulated. Sixty-two percent of the DEGs occurred at both the V1 and V2 time points (Fig. 2B). At 6 months after treatment completion (V5), the Lyme disease transcriptome did not fully return to the baseline relative to controls, with 686 DEGs, 54% (*n* = 373) upregulated and 46% (*n* = 313) downregulated. Partially overlapping clusters were observed for each sample category (V1, V2, V5, and controls) by principal component analysis (PCA) (Fig. 2C).

**TABLE 2:** Number of DEGs with a change of greater than ±1.5-fold, a *P* value of < 0.05, and an FDR of <0.1%

Condition 1 (no. of subjects)	Condition 2 (no. of subjects)	No. of DEGs at:			
		V1^k^	V2^l^	V5^m^	All time points
All Lyme disease (29)	Control (13)	1,235	1,060	686	644
Resolved Lyme disease (15)	Control (13)	1,021	1,090	238	524
Persistent symptoms^a^ (13)	Control (13)	1,358	576	181	641
Persistent symptoms^a^ (13)	Resolved Lyme disease (15)	0	0	0	1^d^
Non-PTLDS^b^ (9)	Resolved Lyme disease (15)	0	0	0	1^e^
PTLDS^c^ (4)	Resolved Lyme disease (15)	1^f^	0	0	3^g^
PTLDS^c^ (4)	Resolved Lyme disease + non-PTLDS*^b^* (24)	0	0	0	2^h^
Disseminated EM (12)	Single EM (17)	0	0	0	0
Seronegative (8)	Seropositive (20)	1^i^	0	0	4^j^
Control (8)	Control (5)	NA^n^	NA	NA	0

a All patients with persistent symptoms following treatment completion.

b Non-PTLDS (persistent symptoms with no functional decline).

c PTLDS (persistent symptoms with functional decline).

d *GPR15*.

e *MIAT*.

f *GPR15*.

g *CCDC163P*, *GRP15*, *ZNF266.*

h *GPR15*, *ZNF266.*

i *HLA-DQB1*.

j *HLA-DQA1*, *HLA-DQB1*, *HLA-DRB5*, *NSA2.*

k Acute Lyme disease diagnosis, pretreatment.

l After 3-week antibiotic treatment.

m At 6 months post-treatment.

n NA, not applicable.

We then calculated differential gene expression between subjects with single versus multiple disseminated EM lesions and between seropositive and seronegative subjects (Table 2). While no DEGs were identified on the basis of single versus multiple lesions, four DEGs were found to be upregulated in seronegative Lyme disease patients relative to those who were seropositive, namely, *HLA-DQA1*, *HLA-DQB1*, *HLA-DRB5*, and *NSA2* (see Fig. S3 in the supplemental material).

### Pathway analyses of the Lyme disease transcriptome.

**FIG 3:**
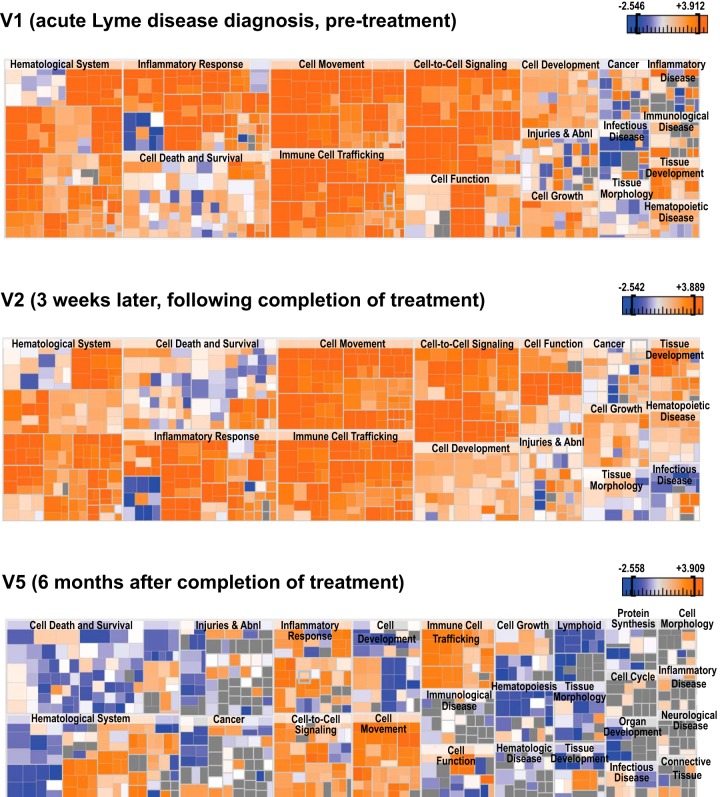
Heat maps of disease and functional categories predicted to be involved in Lyme disease. Shown are heat maps at V1, V2, and V5. The color scheme shown is based on *Z* scores, with activation in orange, inhibition in blue, and undetermined directionality in gray.

Pathway analysis of Lyme disease DEGs revealed predicted activation of inflammatory response, immune cell trafficking, and hematologic system pathways at V1, as expected in the setting of the acute phase of an infection such as Lyme disease (Fig. 2D and 3). However, the same categories also remained activated following the completion of antibiotic treatment and the clinical resolution of symptoms (V2 and V5), with the general pattern of gene expression more inhibitory at V5 (Fig. 3).

Eight, 10, and 4 of the top 10 predicted canonical pathways at V1, V2, and V5, respectively, were directly related to the host immune response (Fig. 2E). The eIF2 signaling pathway, modulating translational initiation and elongation, was found to be significantly downregulated at all three time points (Fig. 2E; see Fig. S4 and Table S2 in the supplemental material). In contrast, TREM1-mediated activation of a Th1 proinflammatory response through upregulation of the factors DAP12, interleukin-6 (IL-6), and IL-12 was prominent at only V1 and V2 (Fig. 2E; see Fig. S4 in the supplemental material). Multiple Toll-like receptors (TLRs) associated with inflammation and apoptosis were also found to be significantly upregulated at V1 and V2 (TLR1, -2, -4, -7, and -8) (see Fig. S4 in the supplemental material).

The most important upstream regulators in Lyme disease at V1 and V2 were found to be proinflammatory cytokines and markers (CSF2, gamma interferon [IFN-γ], IL-1β, IL-6, tumor necrosis factor alpha [TNF-α]), anti-inflammatory cytokines (IL-6, IL-10), the cell surface marker CD40L, transforming growth factor β1, the signal transduction mediator TICAM, the transcriptional regulator NF-κB, and the immunoglobulin complex (Fig. 2F), with TNF-α shown to be a master regulator of eIF2 signaling, TREM1, and TLR pathways (see Fig. S4 in the supplemental material). At V5, the top upstream regulators were predominantly involved in the regulation of gene expression (*MYCN*, *HOX-A7*, *SAT-B1*, *HNF-4A*, *MYC*, *FOS*, and *ELAVL1*) (Fig. 2F).

### Comparison of acute Lyme disease with other infections.

**FIG 4:**
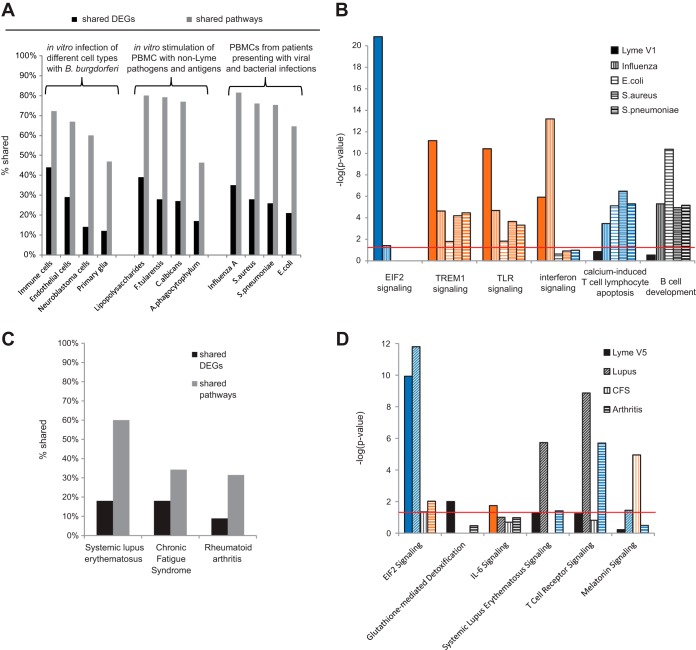
Comparison of the Lyme disease transcriptome with transcriptomes corresponding to other clinical illnesses or cell models. (A) Bar chart of the percentages of overlapping DEGs and canonical pathways between acute Lyme disease at V1 and other infectious diseases or *in vitro* cell culture models of infection. (B) Bar chart of the negative log of the *P* value of the enrichment score corresponding to selected pathways predicted to be involved in acute Lyme disease at V1 compared to other bacterial and viral infections. (C) Bar chart of the percentage of overlapping DEGs and canonical pathways between Lyme disease patients at V5 and three chronic and/or autoimmune syndromes (SLE, CFS, and RA). (D) Bar chart of the negative log of the *P* value of the enrichment score from selected pathways predicted to be involved in Lyme disease 6 months post-treatment (V5) compared to those from other chronic and/or autoimmune syndromes. The color scheme depicted is based on *Z* scores, with activation in orange, inhibition in blue, and undetermined directionality in gray. The red line represents a *P* value significance threshold of 0.05.

We compared our V1 RNA-Seq data, derived from patients with acute Lyme disease, to 12 available, previously published transcriptome data sets from cell culture models of *in vitro* infection or from patients with acute viral and bacterial infections other than Lyme disease (Fig. 4). Unsurprisingly, the greatest overlap in shared DEGs (44%) was observed with *in vitro B. burgdorferi* infection of human PBMCs (44%), followed by infection of human endothelial cells (29%), human neuroblastoma cells (13%), or primary monkey glial cells (11%) (Fig. 4A). We also compared DEGs from acute Lyme disease patients with those corresponding to human PBMCs stimulated *in vitro* by lipopolysaccharides (LPS), infected by the fungal pathogen *Candida albicans*, or infected by two tick-borne bacterial agents, i.e., *Francisella tularensis* (tularemia) and *Anaplasma phagocytophilum* (anaplasmosis). Interestingly, stimulation of PBMCs by LPS (39%) resulted in a greater overlap of shared DEGs than *in vitro* infection with *C. albicans* (27%), *F. tularensis* (28%), or *A. phagocytophilum* (15%) (Fig. 4A). Next, we compared the acute Lyme disease transcriptome at V1 to transcriptomes corresponding to other acute infectious syndromes (Fig. 4A and B). Patients with acute influenza had 35% of their DEGs in common with Lyme disease patients, while they had only 28, 26, and 21% of their DEGs in common with patients with bacteremia due to *Staphylococcus aureus*, *Streptococcus pneumoniae*, and *Escherichia coli*, respectively.

To determine if these infectious diseases have pathways in common, we then compared the top canonical pathways significantly involved in acute Lyme disease at V1 (*P* < 0.05) with those corresponding to other clinical infectious diseases or *in vitro* cell culture models of infection. The greatest number of shared pathways was observed with *S. pneumoniae* bacteremia (82%) and *in vitro F. tularensis* (80%)- and *C. albicans* (78%)-infected cell cultures. Strikingly, downregulation of eIF2 signaling was restricted to Lyme disease and not found in other infectious diseases or *in vitro* cell culture infection models (Fig. 4B). In contrast, TREM1 and TLR pathways were activated in all five infections, whereas upregulation of IFN signaling pathways was predicted in only Lyme disease and influenza. B-cell development and downregulation of calcium-induced T-cell apoptosis, prominent in other viral and bacterial infections, were not found to be significant pathways in acute Lyme disease (Fig. 4B).

### Pathway analysis of Lyme disease patients at 6 months post-treatment and comparison with chronic disease syndromes.

We next compared the PBMC transcriptome from all treated Lyme disease patients at V5 (both resolved illness and persistent symptoms) to publicly available transcriptome data sets from patients diagnosed with chronic illnesses that have some symptoms in common with PTLDS, including chronic fatigue syndrome (CFS), systemic lupus erythematosus (SLE), and rheumatoid arthritis (RA). The percentages of shared DEGs and pathways ranged from 9 to 18% and from 31 to 60%, respectively (Fig. 4C). Inhibition of eIF2 signaling was common to Lyme disease, SLE, and (to a lesser extent) RA (Fig. 4D; see Fig. S5 in the supplemental material). Glutathione-mediated detoxification and IL-6 signaling pathways were found to be specific to Lyme disease patients. No additional differences were revealed by subset analyses of Lyme disease patients manifesting persistent symptoms (non-PTLDS and PTLDS) and patients with these chronic illnesses.

### Comparison of differential gene expression between Lyme disease patients with resolved versus persistent disease.

No DEGs were significantly expressed at any single time point between patients with resolved Lyme disease (*n* = 15) and patients with persistent symptoms, including both non-PTLDS and PTLDS patients (*n* = 13) (Table 2). A single DEG (*GPR15*) was found at V1 when patients with resolved Lyme disease (*n* = 15) were compared with patients with PTLDS (*n* = 4). When all of the time points were combined, a total of four different DEGs overall were identified in Lyme disease patients with persistent symptoms (non-PTLDS and/or PTLDS) compared to those with resolved disease, i.e., *MIAT*, *CCDC163P*, *ZNF266*, and *GPR15*.

## DISCUSSION

We provide the first transcriptome analysis of *B. burgdorferi* infection in Lyme disease patients, revealing a gene signature that persisted for at least 3 weeks following the acute phase of infection and had fewer than 44% DEGs in common with other infectious or noninfectious syndromes. Notably, no differences in gene expression were observed between Lyme disease patients with resolved illness and those with persistent symptoms at 6 months, although larger cohort studies are needed to confirm this finding. The identification of a distinct and sustained transcriptome signature in early Lyme disease may facilitate the development and validation of human gene expression biomarker panels to improve diagnostic testing in the future, in parallel with other published studies investigating cytokine (12) or metabolic (13) biosignatures.

To define the longitudinal transcriptome profile of patients with acute Lyme disease at 0 weeks, 3 weeks, and >6 months, unbiased RNA-Seq analysis was employed with the goal of investigating the molecular basis of early and convalescent-phase Lyme disease. Potential advantages of RNA-Seq relative to microarrays include detection of low-abundance transcripts, a broader dynamic range in detecting fold changes in gene expression, unbiased detection of novel isoforms and transcripts, and elimination of hybridization-based limitations such as background noise, saturation, and probe redundancy (14). However, the utility of RNA-Seq data is dependent on a number of factors, including the number and quality of samples, sequencing depth, and designated thresholds for gene expression and differential analyses. In this study, we estimated the statistical power as 98% when analyzing samples at all three time points combined, 78% for samples collected at a single time point, and only 62% when considering a stratification of the Lyme disease cohort according to PTLDS status, serology, or the presence of disseminated lesions (15).

The finding of a profound and sustained change in the transcriptome of acute Lyme disease patients refutes the idea that treatment and resolution of the infection result in a prompt return to a transcriptional baseline, as typically seen in the acute phase of other infections (16). In addition, failure to return to a transcriptome baseline cannot be accounted for solely by patients with persistent symptoms, given that no DEGs were found comparing Lyme disease patients with resolved illness to those with persistent symptoms (Table 2). Persistent transcriptional changes may be characteristic of not only Lyme disease but also a number of other infections. For example, viral clearance in hepatitis C patients did not result in normalization of the baseline transcriptome (17). To our knowledge, this is first time that sustained changes in the human host transcriptome have been reported for a bacterial infection after treatment with appropriate antibiotics. Persistence of such a signature for at least 3 weeks following infection suggests that a clinical diagnostic test for acute Lyme disease based on host gene expression is feasible. Such a test would also directly address the current diagnostic gap created by the “window period” between acute Lyme disease infection and the subsequent appearance of detectable antibody.

Infection by *B. burgdorferi* drives a complex immune response with robust inflammation and overt clinical signs and symptoms in early stages of the disease (7). The eIF2 signaling pathway, found to be downregulated here during all stages of Lyme disease, plays a central role in protein synthesis in response to cellular stress (18). Intracellular bacterial pathogens such as *Legionella pneumophila* encode effectors that actively disrupt and downregulate the eIF2 signaling pathway (19). However, *Borrelia* spirochetes are not known to enter cells during infection *in vivo*, nor are they thought to express such effectors (20). Consistent with a previous report (21), the eIF2 pathway in this study was also found to be downregulated in SLE as well as PTLDS patients. Inhibitors of the eIF2 pathway have been reported as potential therapeutic drugs for inflammatory bowel disease, and further studies are needed to assess whether eIF2 inhibitors may constitute potential targets for inflammatory sequelae of Lyme disease (22). Nevertheless, it remains to be determined whether inhibition of the eIF2 pathway in Lyme disease patients is caused directly by *Borrelia*-mediated immune dysregulation or is strictly a host response mechanism to limit tissue injury.

The prominent TREM1 signaling in acute Lyme disease observed here is consistent with previously published *in vitro* gene expression data of *B. burgdorferi* infection of human neural and primary monkey glial cell lines (23). TREM1 acts as an amplifier of the immune and inflammatory response *in vivo* (24), and modulation of TREM1 has been shown to impact a number of inflammatory conditions, including septic shock, and acute dengue virus infection (25, 26). Our data also showed upregulation of more TLRs (TLR2, TLR4, TLR7, and TLR8) in acute Lyme disease than previously described (27). This broad upregulation is likely to be indirect, reflecting a general increase in TLR regulatory activity rather than direct association of TLRs with *B. burgdorferi* proteins. In the present study, TNF-α was predicted to be a common upstream regulator of the eIF2, TREM1, and TLR signaling pathways. Notably, anti-TNF-α therapy has been proposed to reduce inflammation in the Jarisch-Herxheimer response to *Borrelia recurrentis* infection (28), and treatment was previously reported to be clinically efficacious in 4/4 patients with antibiotic-refractory Lyme arthritis (29).

Comparisons with 15 previously published transcriptome data sets found that the greatest overlap of DEGs (44%) was with the transcriptome of PBMCs stimulated with *B. burgdorferi* in vitro. Although this observation is to be expected, given the same infectious agent and cell type, the only partial overlap likely reflects differences between *in vivo* or *in vitro B. burgdorferi* infections and underscores the critical importance of analyzing “real-life” clinical samples from patients in studies of disease pathogenesis. Given the lymphocytic infiltrates characteristic of Lyme disease, in contrast with the suppurative lesions common to many bacterial infections (1), it is perhaps not surprising that the percentage of DEGs in Lyme disease patients also found in patients with acute influenza was greater than that of DEGs also found in patients with other bacterial infections. Among bacterial infections, infection with *S. pneumoniae* had the highest number of top canonical pathways in common with acute Lyme disease, consistent with similarities in virulence factors shared by *S. pneumoniae* and *B. burgdorferi*, such as lipoproteins, that produce shared IgM-mediated immunological responses (30).

Importantly, Lyme disease patients did not show any changes in the calcium-dependent T-cell apoptosis pathway, in contrast to the marked downregulation observed in other bacterial and viral diseases (Fig. 4B). In addition, an absence of significant DEGs linked to B-cell development in Lyme disease relative to other infections was observed. These findings suggest that Lyme disease may be associated with a smaller proportion of B and T cells in peripheral blood than other diseases. Interestingly, suppression of long-lived humoral responses has been observed in a mouse model of *Borrelia* infection (31). The absence of DEGs corresponding to B-cell maturation may also potentially explain why prior infection with *B. burgdorferi* is associated with a serological response yet does not appear to confer immunity to reinfection. Certain alleles of HLA genes have been previously reported to be associated with serological responses to Lyme disease infection (32). Here we found that upregulation of certain HLA genes (*HLA-DQA1*, *HLA-DQB1*, *HLA-DRB5*) is associated with seronegativity in Lyme disease and may thus constitute potential diagnostic biomarkers for seronegative patients.

Following the acute phase of infection, recent treatment trials among patients with EM have estimated that approximately 10 to 20% of patients treated for Lyme disease experience lingering symptoms that may progress to PTLDS, although the incidence can be as high at 50% (4). The pathogenetic mechanisms of PTLDS remain unknown, but autoantigens and/or central nervous system sensitization have been postulated to play a role (10, 33–35). In our study, the relatively large proportion of post-treatment Lyme disease patients with persistent symptoms of fatigue, widespread musculoskeletal pain, and/or cognitive dysfunction (13 [46.4%] of 28) can be potentially accounted for by more stringent enrollment criteria at the time of presentation (requiring the presence of EM and concurrent influenza-like symptoms rather than EM alone). This may have resulted in the selection of patients with more severe disease and thus with an increased likelihood of persistent symptoms (36). Of note, according to the proposed formal case definition for PTLDS, which requires a functional decline in patients in addition to lingering symptoms, only 4 (14.3%) of our 28 patients met all of the criteria, within the range of the 10 to 20% frequency reported in the literature (4).

Notably, Lyme disease at 6 months post-treatment (V5) had 60 and 31% of their predicted pathways overall in common with SLE and RA, respectively. Circulating immune complexes have been identified as features common to all three conditions (37, 38). Symptoms of fatigue and cognitive impairment occur in a variety of chronic syndromes, including SLE, CFS, and PTLDS. Although some pathways were common to Lyme disease at V5 and CFS, melatonin signaling, prominent in CFS, was not predicted to be involved in Lyme disease (Fig. 4D). As melatonin is a hormone that regulates the circadian rhythms of the sleep-wake cycle and thus is strongly linked to fatigue, the absence of increased melatonin signaling suggests that the fatigue in Lyme disease patients with persistent symptoms is related to a different mechanism. Overall, our results, showing only 18% of the DEGs and 34% of the pathways common to CFS and Lyme disease, are consistent with a proteomic study of cerebrospinal fluid that clearly discriminates between the two conditions (39).

Transcriptome analysis of Lyme disease patients with persistent symptoms (non-PTLDS and/or PTLDS) versus those with resolved illness revealed an absence of DEGs at each of the three time points, with the sole exception of a single gene (*GPR15*), which was upregulated at V1 in PTLDS patients relative to controls. Possible explanations for the overall lack of observed differences include (i) lack of statistical power from low sample numbers, (ii) sampling at designated time points instead of during periods of peak symptomatology, and (iii) that transcriptome profiling of PBMCs in blood is insufficient to discriminate between Lyme disease patients with persistent symptoms and those with resolved illness. Larger studies with increased sampling resolution are needed to establish whether there are indeed any detectable differences in gene expression between these two groups.

## MATERIALS AND METHODS

### Patient information.

Patient enrollment, collection of clinical data and biological samples, and analysis of clinical samples by transcriptome profiling were done under protocols approved by the Institutional Review Boards of Johns Hopkins University and the University of California, San Francisco. Written informed consent was received from all participants prior to inclusion in this study.

All 29 participants with Lyme disease included in this study presented with a physician-documented EM rash of ≥5 cm and concurrent influenza-like symptoms that included at least one of the following; fever, chills, fatigue, headache, and/or new muscle or joint pains. At the time of enrollment, all of the participants with Lyme disease were treatment naive and subsequently underwent 3 weeks of doxycycline therapy between the first and second follow-up visits. All 29 subjects with Lyme disease were enrolled at the same geographic location (an outpatient clinic in Maryland) in a single season, from 1 May to 23 November 2009, with follow-up visits 3 weeks and 6 months after the first visit. Controls were matched by age and gender and enrolled from the same physician practice as case participants and across different seasons to account for seasonal variations in the transcriptome. Two-tier antibody testing for Lyme disease by whole-cell sonicate enzyme immunoassay, followed by IgM/IgG Western immunoblot assays, was performed for all patients and controls by a clinical reference laboratory (Quest Diagnostics). Seropositivity was assessed according to established CDC criteria (40) by the investigators (A.R. and J.A.) who enrolled and provided clinical care for the patients enrolled in this study. All control subjects were required to have a negative Lyme disease antibody test in order to be enrolled in this study. We screened both patients and controls prior to enrollment for a history of chronic fatigue, fibromyalgia, autoimmune, immunodeficiency, neurologic, psychiatric, and malignancy disorders, in which case they were excluded from the study. Prospective case patients and controls were also excluded if they had a prior documented history of Lyme disease and/or if they had previously received the Lyme disease vaccine.

Controls were enrolled primarily during the winter and spring seasons, while most Lyme disease patients were enrolled in summer during the peak season for tick bites, a difference that was statistically significant (*P* < 0.03) (Table 1). Nonetheless, the differences in seasonal sampling did not result in gene expression bias, as shown by the absence of seasonal clustering by PCA of the overall gene expression of the 13 controls (see Fig. S2b in the supplemental material). In addition, an intragroup comparison of eight controls sampled during the winter and five controls sampled during other seasons did not yield any significant DEGs (Table 2).

PBMCs from whole-blood samples at V1 (the acute phase of infection, prior to initiation of antibiotic treatment), V2 (3 weeks later, at the time of treatment completion), and V5 (6 months following treatment completion) were analyzed in this study (Fig. 1A). V2 and V5 were specifically chosen for analysis because fever and rash from acute Lyme disease typically resolve by completion of treatment (V2), while chronic persistent symptoms are clinically apparent after 6 months (V5).

The presence of persistent symptoms in Lyme disease patients at V5 was assessed by using a standardized case definition proposed by the Infectious Diseases Society of America (6, 11) that incorporates the presence of at least one of the following: new-onset fatigue, widespread musculoskeletal pain, or cognitive dysfunction. For a diagnosis of PTLDS, patients were also required to have a composite score of ≤45.00 on four subscales of the SF-36, a measurement of health-related quality of life (6) (Fig. 1B). The chi-square test was used to evaluate the statistical significance of differences between independent samples in one or more categorical variables, while Welch’s *t* test was used for continuous variables.

### Sample processing.

PBMCs were isolated from fresh whole blood with Ficoll (Ficoll-Paque Plus; GE Healthcare), and total RNA was extracted from 10^7^ PBMCs with TRIzol reagent (Life Technologies). mRNA was isolated with the Oligotex mRNA minikit (Qiagen). The ScriptSeq RNA-Seq library preparation kit (Epicentre) was used to generate RNA-Seq libraries according to the manufacturer’s protocol. Libraries were sequenced as 100-bp paired-end runs on a HiSeq 2500 (Illumina). One hundred samples from the first cohort (29 patients at three time points and 13 control subjects, matched by age, sex, and geography) were mixed and blindly processed in three batches. Three samples, 01-36_V2, 01-42_V2, and 01-51_V1, were not included in the pooled analysis because of insufficient read counts and transcriptome coverage (see Fig. S1 in the supplemental material). No batch effect was observed by PCA of the global expression of all 25,278 genes (see Fig. S6 in the supplemental material).

### Next-generation sequencing data analysis.

Paired-end reads were mapped to the human genome (hg19), followed by annotation of exons and calculation of FPKM (fragments per kilobase of exon per million fragments mapped) values for all 25,278 expressed genes with version 2 of the TopHat-Cufflinks pipeline (41). Differential expression of genes was calculated by using the variance modeling at the observational level transformation (42), which applies precision weights to the matrix count, followed by linear modeling with the Limma package (43). Genes were considered to be differentially expressed when the change was greater than ±1.5-fold, the *P* value was <0.05, and the adjusted *P* value (or false-discovery rate, FDR) was <0.1% (44). Pathway and network analyses of the transcriptome data were performed with Ingenuity Pathway Analysis (IPA) software (Qiagen) (45). The molecule activity predictor tool in the IPA software was used to predict the upstream and/or downstream activation or inhibition of a given pathway. The *P* value of the enrichment score was used to evaluate the significance of the overlap between observed and predicted gene sets, while the activation *Z* score was used to assess the match between observed and predicted patterns of upregulation and downregulation. The statistical significance of the difference in gene expression levels was determined with Welch’s *t* test for independent samples by two-group comparisons. The statistical power for the transcriptome study was determined according to the algorithm developed by Hart et al. (15), with the use of a generalized linear model on normalized FPKM data instead of a negative binomial distribution on raw gene count data. The generalized linear model has been reported to be more reliable for differential analysis of data sets with small sample sizes (41, 43).

### Comparison of RNA-Seq and microarray data.

Microarray transcriptome data were downloaded from public servers (http://www.ncbi.nlm.nih.gov/geo) and include expression sets GSE12108, GSE2405, GSE42606, GSE8650, GSE6269, GSE6092, GSE14577, and GSE15573 (46–52). Raw data were extracted and preprocessed by using the Robust Multichip Average algorithm (53). Differential expression was calculated with the Limma package (43), which is applicable for analysis of both RNA-Seq and microarray data (42, 43). Genes were considered to be differentially expressed when the change was greater than ±1.5-fold, the *P* value was <0.05, and the FDR was <0.1%, in accordance with conventional thresholds (44). Microarray data were not available for one study of *in vitro B. burgdorferi* infection (23), so tables of DEGs were used as provided instead, incorporating a change of greater than ±1.5-fold as a threshold cutoff for differential expression.

### Data availability.

All of the transcriptome data obtained in this study have been submitted to the Gene Expression Omnibus data repository under accession number GSE63085.

## SUPPLEMENTAL MATERIAL

Table S1  Clinical presentations of Lyme disease patients with resolved illness versus those with persistent symptoms. Table S1, XLSX file, 0.01 MB

Table S2  List of canonical pathways predicted to be involved in Lyme disease. Table S2, XLSX file, 0.04 MB

Figure S1  Next-generation sequencing read counts and human transcriptome coverage. (A) Total read counts per sample. (B) Coverage of the transcriptome as expressed for each sample as the percentage of genes for which FPKM values are nonzero. Samples were processed in three batches. Two samples indicated by a red bar in panel A were not included in this study, as they had a read count of <10,000 sequences, too low to obtain a representative transcriptome coverage. One sample in panel B, represented by a red bar, was not included in this study, as the coverage of its transcriptome was very low (3.44%). Download Figure S1, AI file, 1.2 MB

Figure S2  PCA of the global gene expression of 13 matched controls. Dot colors indicate the sampling seasons, with spring in yellow, summer in red, autumn in green, and winter in blue. Overall gene expression is not driven by sampling season, as demonstrated by the absence of clustering based on patients sampled in similar seasons. The asterisk represents a control subject appearing to be a PCA outlier in Fig. 2C. Download Figure S2, AI file, 0.1 MB

Figure S3  Expression levels of four DEGs between seropositive and seronegative Lyme disease patients. Red asterisks represent significant *P* values for differences between seropositive and seronegative Lyme disease patients at all of the time points examined (*, *P* <0.05; **, *P* <0.01; ***, *P* <0.001 [Welch’s *t* test]). Download Figure S3, AI file, 0.2 MB

Figure S4  Illustration of acute Lyme disease pathways predicted at V1. The eIF2 (A), TREM1 (B), and TLR (C) signaling pathways are represented, highlighting the transcripts, proteins, and cofactors found to be differentially expressed or predicted to be involved in Lyme disease relative to controls. (D) Mechanistic network driven by a top upstream regulator at V1 and V2, TNF, driving inflammation and regulating downstream eIF2, TREM1, and TLR signaling pathways (red, transcript upregulation; green, transcript downregulation; orange, predicted activation; blue, predicted inhibition; brown, findings inconsistent with state of downstream molecule; gray, effect not predicted; yellow, potential Lyme disease biomarker). Download Figure S4, PDF file, 1.2 MB

Figure S5  Illustration of Lyme disease pathways predicted at 6 months post-treatment (V5). The eIF2 signaling (A), glutathione-mediated detoxification (B), and IL-6 signaling (C) pathways are represented, highlighting the transcripts, proteins, and cofactors found to be differentially expressed or predicted to be involved in Lyme disease patients relative to controls (red, transcript upregulation; green, transcript downregulation; orange, predicted activation; blue, predicted inhibition; brown, findings inconsistent with state of downstream molecule; gray, effect not predicted; yellow, potential Lyme disease biomarker). Download Figure S5, PDF file, 4.9 MB

Figure S6  PCA of the expression of 25,278 genes according to batch number. No batch effect was observed. Abbreviations: PC1, principal component axis 1; PC2, principal component axis 2. Download Figure S6, AI file, 2.3 MB
