# Supplementary material for: Longitudinal Transcriptome Analysis Reveals a Sustained Differential Gene Expression Signature in Patients Treated for Acute Lyme Disease
Source: mBio. 2016 Feb 12;7(1):e00100-16. doi: 10.1128/mBio.00100-16 (PMC4791844; doi:10.1128/mBio.00100-16)
Supplement: Figure S4 — Illustration of acute Lyme disease pathways predicted at V1. The eIF2 (A), TREM1 (B), and TLR (C) signaling pathways are represented, highlighting the transcripts, proteins, and cofactors found to be differentially expressed or predicted to be involved in Lyme disease relative to controls. (D) Mechanistic network driven by a top upstream regulator at V1 and V2, TNF, driving inflammation and regulating downstream eIF2, TREM1, and TLR signaling pathways (red, transcript upregulation; green, transcript downregulation; orange, predicted activation; blue, predicted inhibition; brown, findings inconsistent with state of downstream molecule; gray, effect not predicted; yellow, potential Lyme disease biomarker). Download [file mbo001162702sf4.pdf]

A

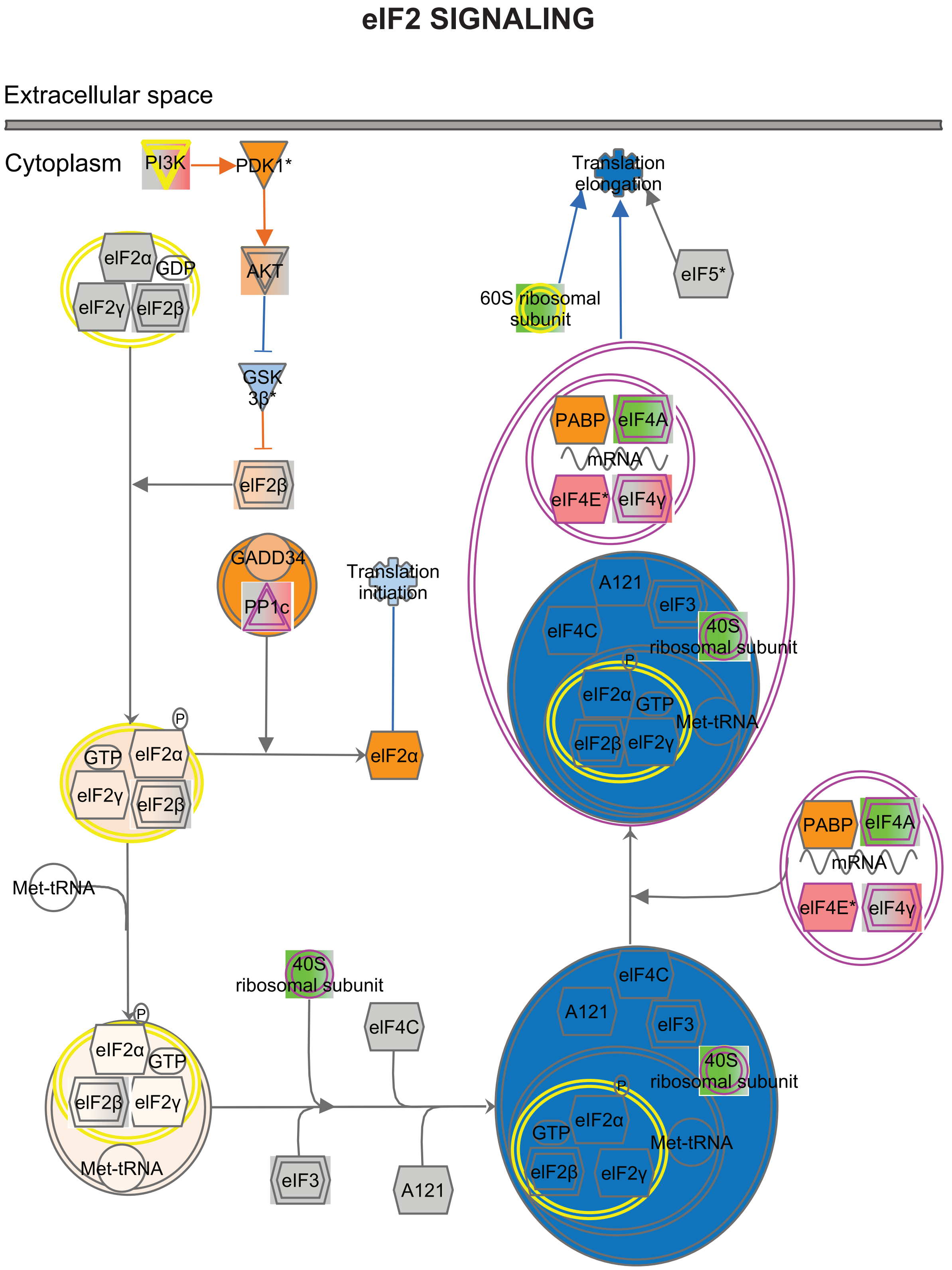

Supplementary Figure 4. Illustration of acute Lyme disease pathways predicted at V1. The (a) eIF2, (b) TREM1, and (c) TLR signaling pathways are represented, highlighting the transcripts, proteins and co-factors found to be differentially expressed or predicted to be involved in Lyme disease relative to controls. (d) Mechanistic network driven by a top upstream regulator at V1 and V2, TNF, driving inflammation and regulating downstream eIF2, TREM1 and TLR signaling pathways (red = transcript up-regulation, green = transcript down-regulation, orange = predicted activation, blue = predicted inhibition, brown = findings inconsistent with state of downstream molecule, gray = effect not predicted, yellow = potential Lyme disease biomarker).

B

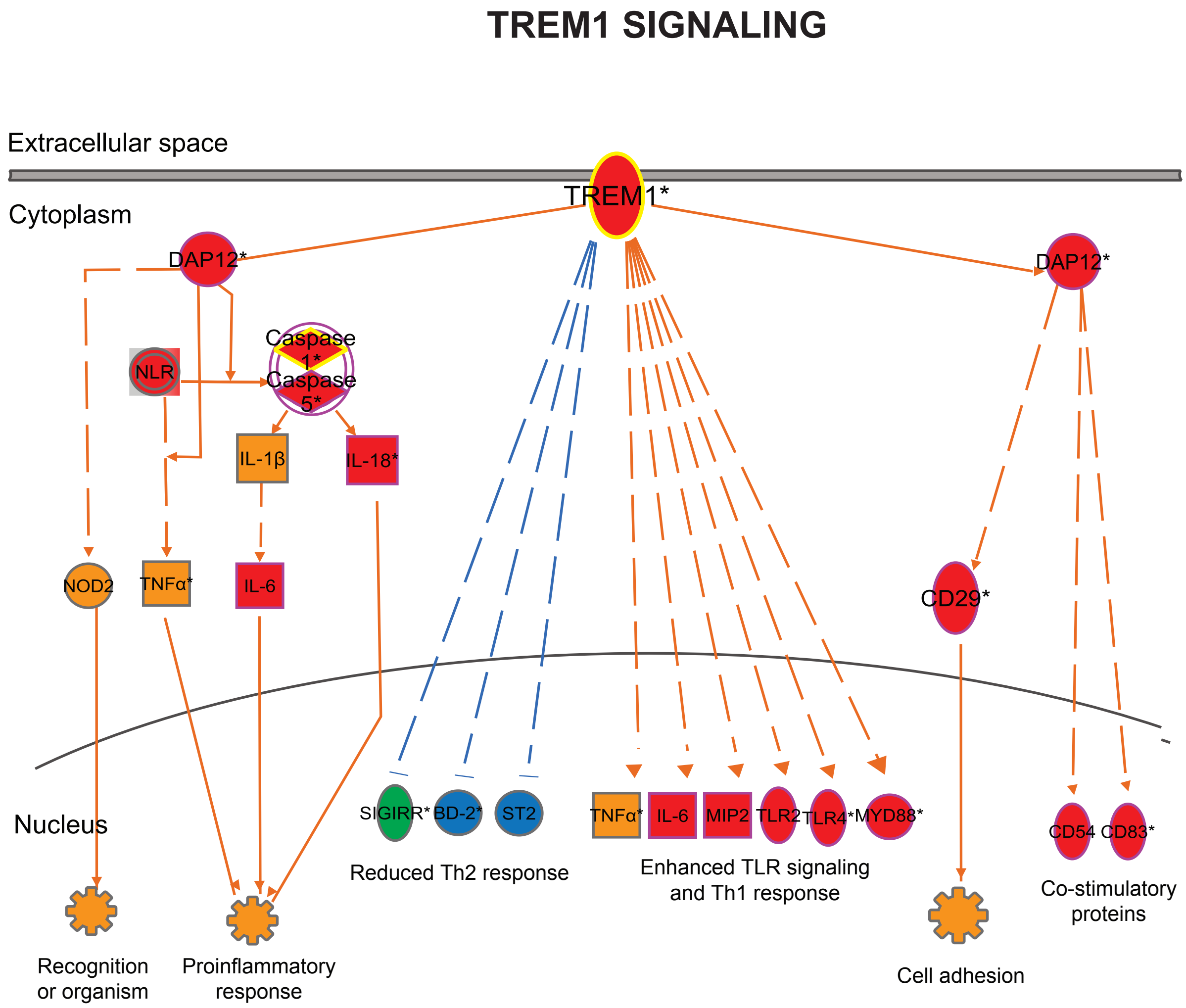
TREM1 SIGNALING
Extracellular space
Cytoplasm
Nucleus
TREM1*
DAP12*
DAP12*
NLR
Caspase 1*
Caspase 5*
IL-1β
IL-18*
NOD2
TNFα*
IL-6
CD29*
SIGIRR*
BD-2*
ST2
TNFα*
IL-6
MIP2
TLR2
TLR4*
MYD88*
CD54
CD83*
Recognition or organism
Proinflammatory response
Reduced Th2 response
Enhanced TLR signaling and Th1 response
Cell adhesion
Co-stimulatory proteins

C

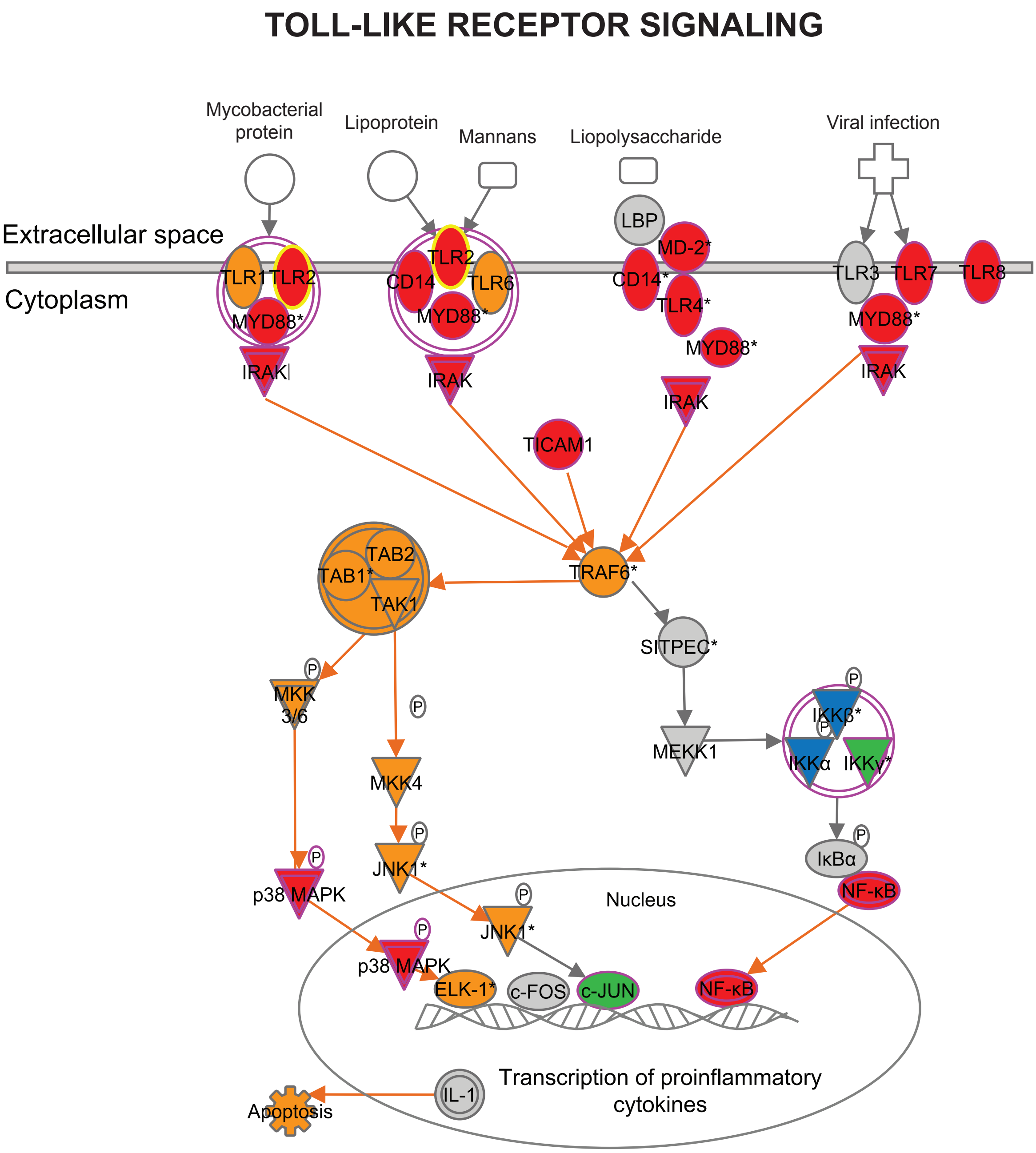

TOLL-LIKE RECEPTOR SIGNALING
Mycobacterial protein
Lipoprotein
Mannans
Liopolysaccharide
Viral infection
Extracellular space
Cytoplasm
TLR1
TLR2
MYD88*
IRAK
CD14
TLR6
LBP
MD-2*
CD14*
TLR4*
TLR3
TLR7
TLR8
TICAM1
TAB2
TAB1*
TAK1
TRAF6*
SITPEC*
MKK 3/6
MKK4
MEKK1
IKKβ*
IKKα
IKKγ*
JNK1*
IκBα
NF-κB
p38 MAPK
Nucleus
ELK-1*
c-FOS
c-JUN
Transcription of proinflammatory cytokines
IL-1
Apoptosis
P

D

TNF-DRIVEN MECHANISTIC NETWORK

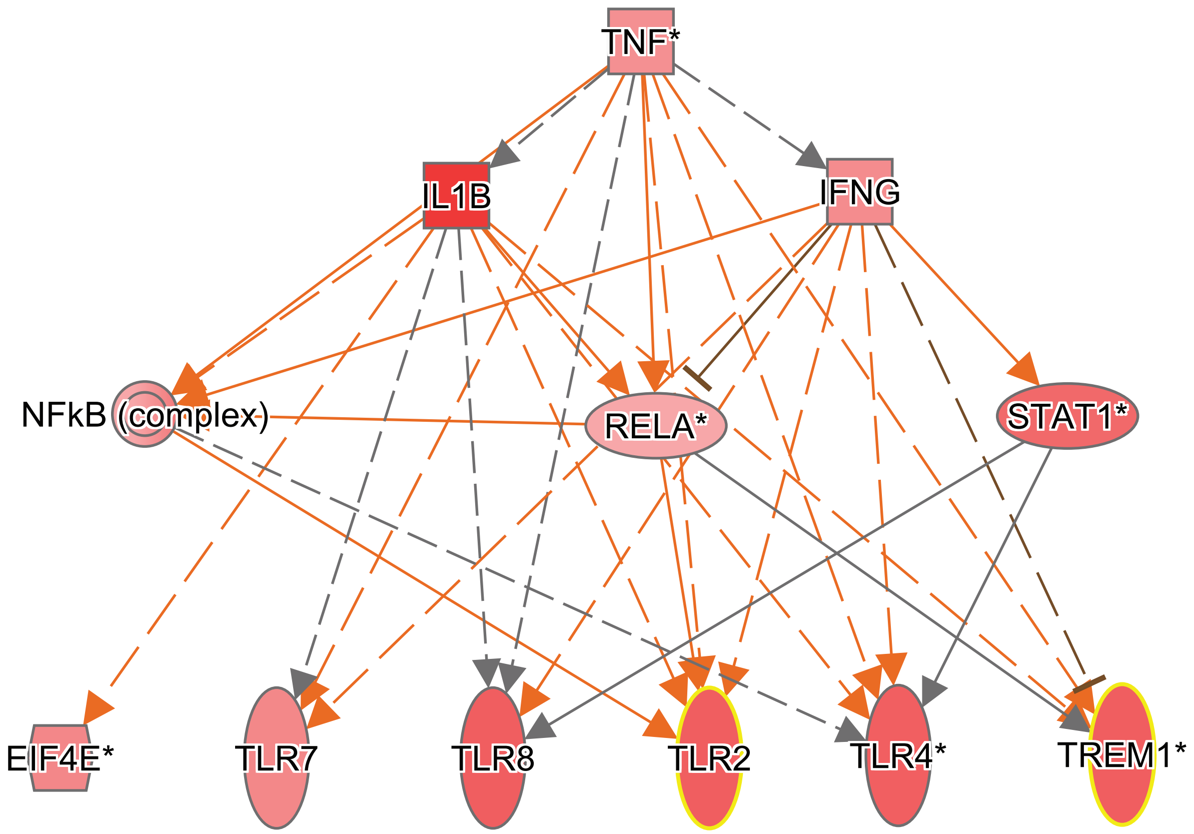

LEGEND

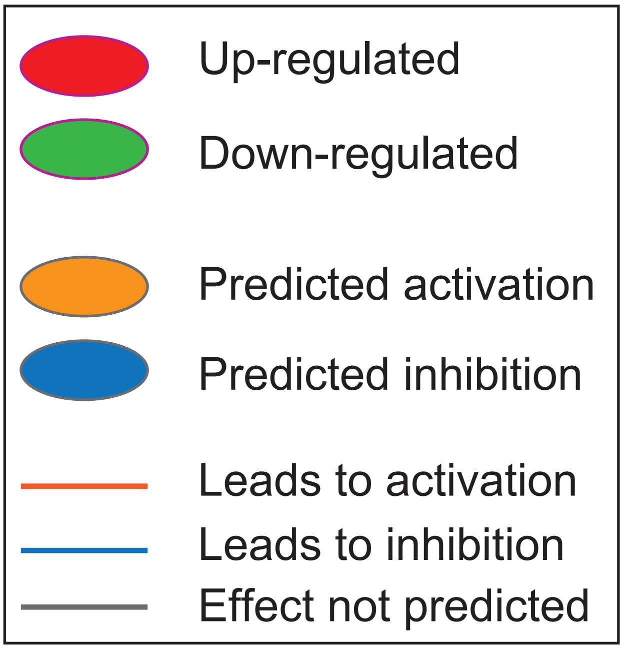
Up-regulated
Down-regulated
Predicted activation
Predicted inhibition
Leads to activation
Leads to inhibition
Effect not predicted
