# Supplementary material for: Longitudinal Transcriptome Analysis Reveals a Sustained Differential Gene Expression Signature in Patients Treated for Acute Lyme Disease
Source: mBio. 2016 Feb 12;7(1):e00100-16. doi: 10.1128/mBio.00100-16 (PMC4791844; doi:10.1128/mBio.00100-16)
Supplement: Figure S5 — Illustration of Lyme disease pathways predicted at 6 months post-treatment (V5). The eIF2 signaling (A), glutathione-mediated detoxification (B), and IL-6 signaling (C) pathways are represented, highlighting the transcripts, proteins, and cofactors found to be differentially expressed or predicted to be involved in Lyme disease patients relative to controls (red, transcript upregulation; green, transcript downregulation; orange, predicted activation; blue, predicted inhibition; brown, findings inconsistent with state of downstream molecule; gray, effect not predicted; yellow, potential Lyme disease biomarker). Download [file mbo001162702sf5.pdf]

A

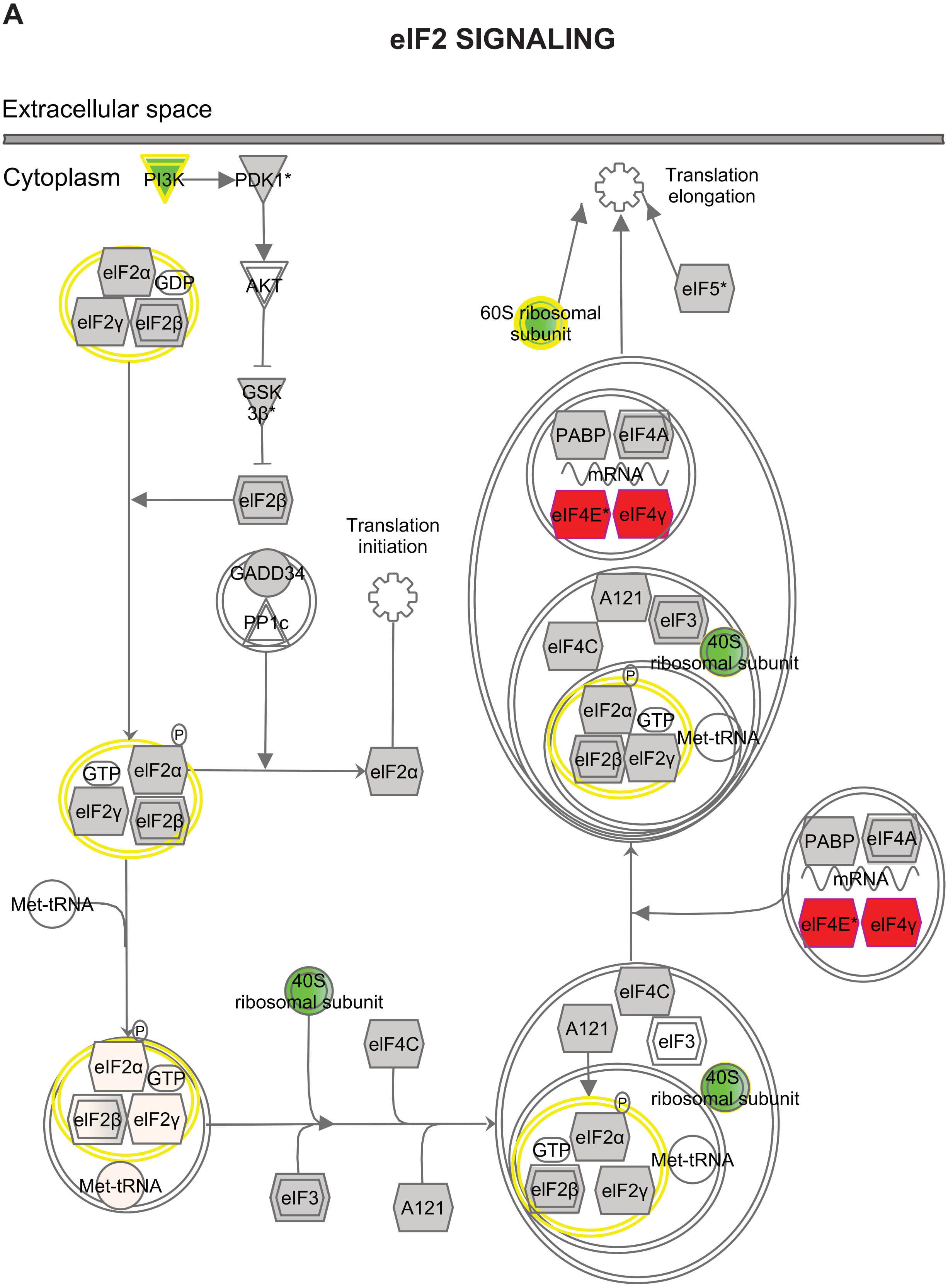

Supplementary Figure 5. Illustration of Lyme disease pathways predicted at 6 months post-treatment (V5). The (a) eIF2 signaling, (b) Glutathione-mediated detoxification, and (c) IL-6 signaling pathways are represented, highlighting the transcripts, proteins and co-factors found to be differentially expressed or predicted to be involved in Lyme disease relative to controls (red = transcript up-regulation, green = transcript down-regulation, orange = predicted activation, blue = predicted inhibition, brown = findings inconsistent with state of downstream molecule, gray = effect not predicted, yellow = potential Lyme disease biomarker).

B

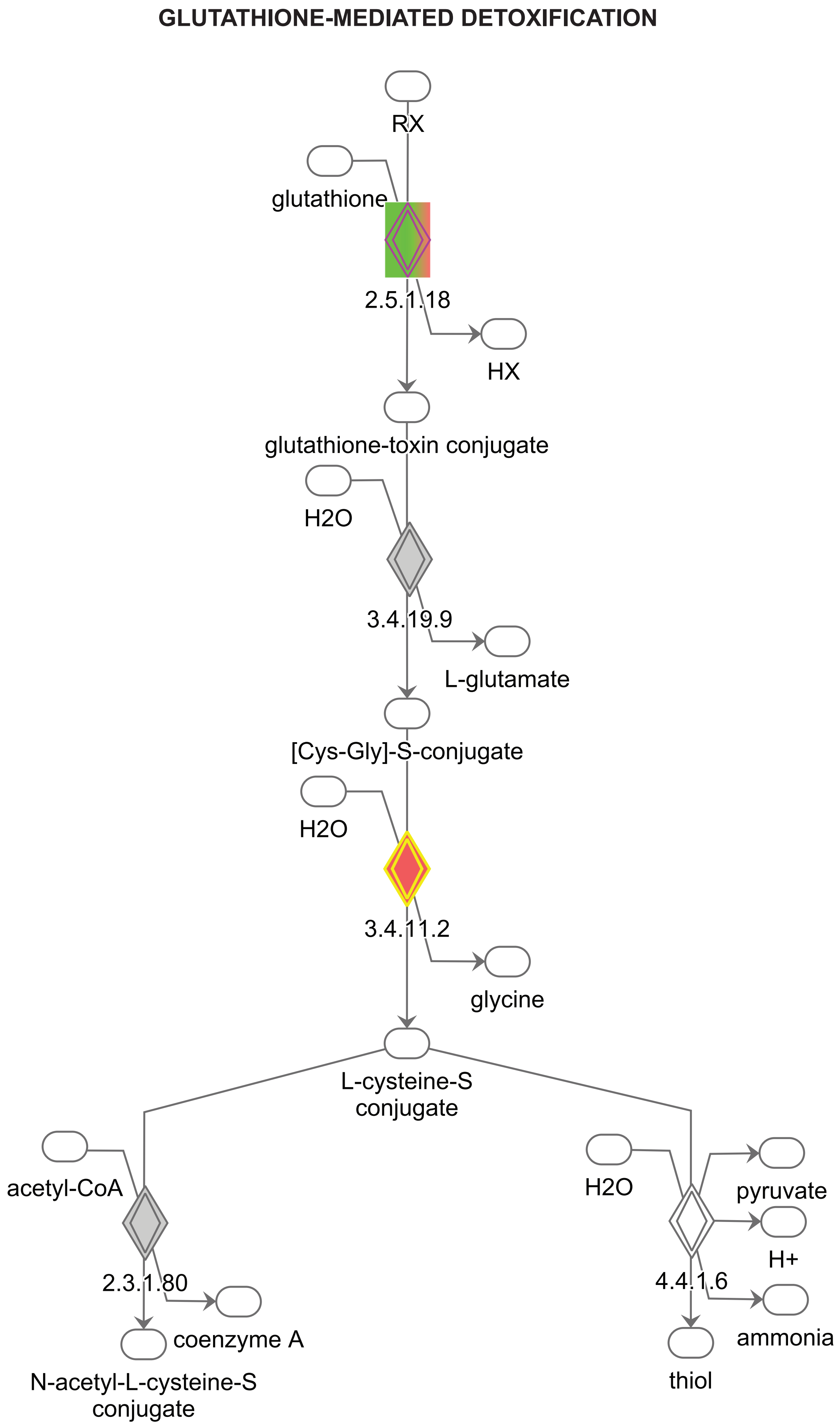
GLUTATHIONE-MEDIATED DETOXIFICATION
RX
glutathione
2.5.1.18
HX
glutathione-toxin conjugate
H2O
3.4.19.9
L-glutamate
[Cys-Gly]-S-conjugate
H2O
3.4.11.2
glycine
L-cysteine-S conjugate
acetyl-CoA
2.3.1.80
coenzyme A
N-acetyl-L-cysteine-S conjugate
H2O
pyruvate
H+
4.4.1.6
ammonia
thiol

C

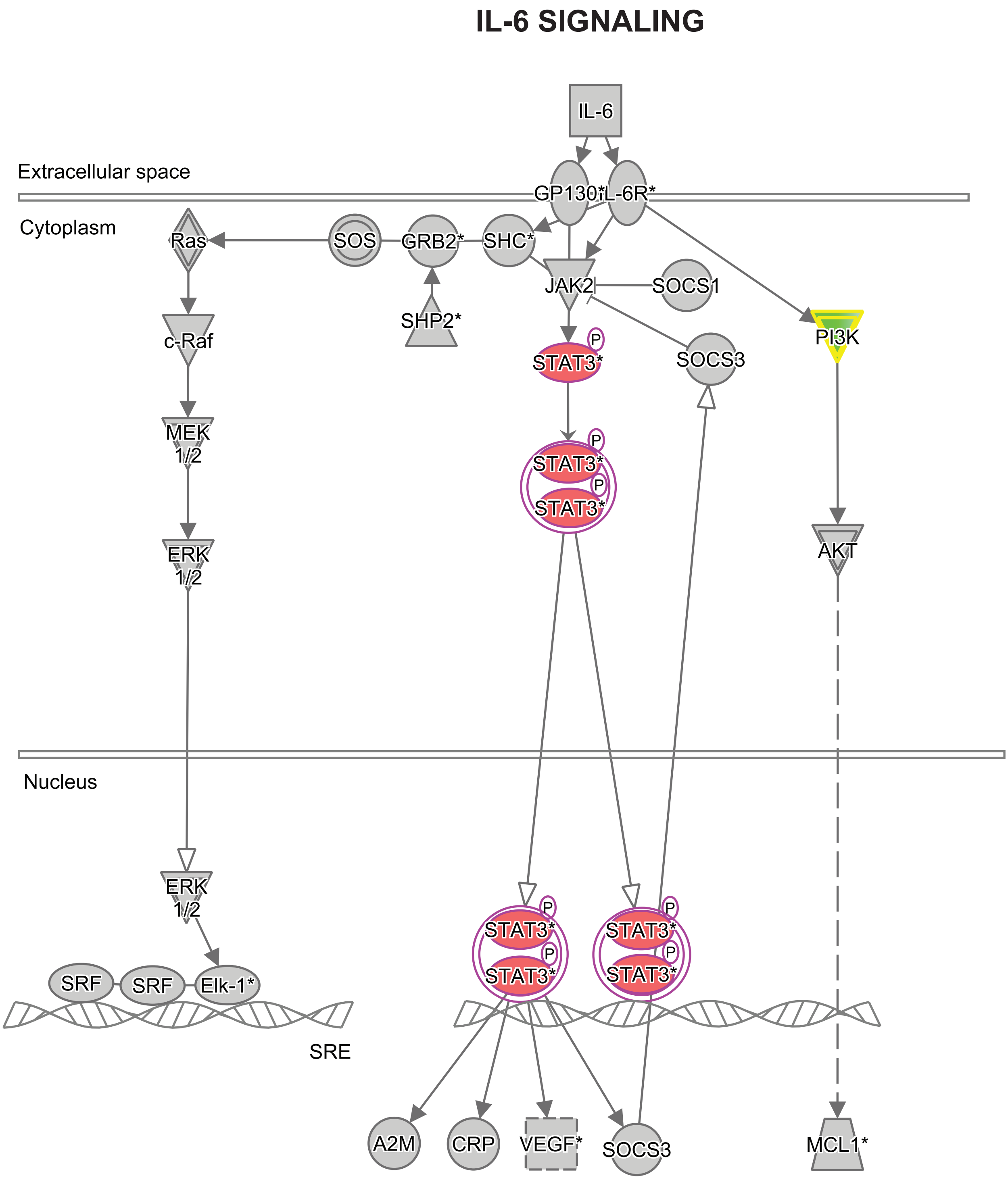
IL-6 SIGNALING
IL-6
Extracellular space
GP130*
IL-6R*
Cytoplasm
Ras
SOS
GRB2*
SHC*
JAK2
SOCS1
SHP2*
c-Raf
STAT3*
P
SOCS3
PI3K
MEK 1/2
ERK 1/2
AKT
Nucleus
SRF
SRF
Elk-1*
SRE
A2M
CRP
VEGF*
SOCS3
MCL1*

LEGEND

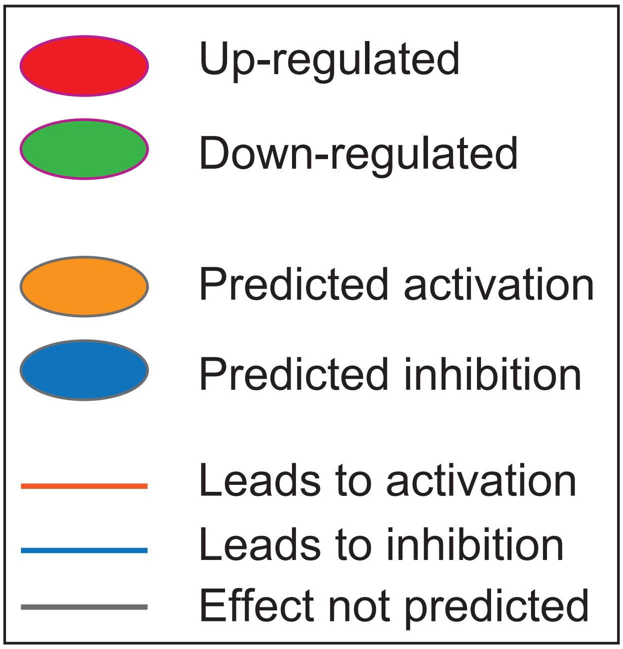
Up-regulated
Down-regulated
Predicted activation
Predicted inhibition
Leads to activation
Leads to inhibition
Effect not predicted
